# Elbasvir/grazoprevir treatment in an HCV-infected peritoneal dialysis patient

**DOI:** 10.1080/0886022X.2020.1753073

**Published:** 2020-04-17

**Authors:** Jin Chen, Yi Li, Guisen Li, Pu Lei

**Affiliations:** a Renal Department and Institute of Nephrology, Sichuan Academy of Medical Sciences & Sichuan Provincial People’s Hospital, Chengdu, China; b School of Medicine, University of Electronic Science and Technology of China, Chengdu, China

**Keywords:** Elbasvir, grazoprevir, hepatitis C virus, peritoneal dialysis

## Abstract

Hepatitis C virus (HCV) infection is known to affect long-term patient survivals. Elbasvir/grazoprevir (EBR/GZR) has shown a high cure rate in hemodialysis patients with HCV infection. However, the combination is rarely used in peritoneal dialysis patients. Herein, we report a case of successful treatment with EBR/GZR in a peritoneal dialysis patient with HCV genotype 1 b infection. A 54-year-old woman on peritoneal dialysis（PD）with HCV genotype 1 b infection had been received EBR (100 mg) and GZR (50 mg) once daily for 12 weeks. Hepatitis C virus RNA was undetectable 4 weeks after the treatment. She achieved a sustained virological response at 12 weeks after the end of treatment. Only fatigue was reported as side effect during the treatment. Thus, elbasvir/grazoprevir was effective and safe in this PD patient with HCV genotype 1 b infection.

Hepatitis C virus (HCV) infection is highly prevalent in patients receiving renal replacement therapy [1] and has been reported in 10–15% of dialysis patients worldwide [2]. Further, it increases hospitalization and mortality risks in dialysis patients [3]. Peritoneal dialysis (PD) is a well-established technique of renal replacement therapy for patients with CKD G5. More than 272,000 patients receive PD worldwide, about 11% of the total dialysis population [4]. Some reports showed that the prevalence of anti-HCV in PD patients was 8–8.6% [5,6]. Kidney Disease Improving Global Outcomes (KDIGO) recommends all HCV-infected chronic kidney disease (CKD) patients should be evaluated for antiviral therapy. If the benefits of antiviral treatment outweigh harms, patient with CKD G4-G5D should be treated with a ribavirin-free direct-acting antivirals (DAAs) based regimen [1]. Very few anti-HCV therapy studies included PD patients, so that there is no enough evidence to support specific DAA regimens in PD patients. Experts in KIDIGO believe it is reasonable to follow proposed regimens for hemodialysis (HD) patients. Although EBR/GZR and Glecaprevir/pibrentasvir are recommended to HD patients by guideline, but up to now, only EBR/GZR is available in China.

Elbasvir/grazoprevir (EBR/GZR) is a fixed-dose combination treatment for HCV genotype 1 or 4 infection. The efficacy and safety of EBR/GZR in CKD patients, including those on HD, has been reported in studies [7–10]. However, to our knowledge, there are no reports on EBR/GZR use in HCV-infected PD patients. Herein, we report a case of successful treatment of HCV genotype 1 b infection in a PD patient by using EBR/GZR.

## Case report

A 54-year-old Chinese woman with chronic HCV infection and CKD G5D on PD was referred for anti-HCV therapy. Recent abdominal computed tomography scan and transient liver elastography (Fibroscan) indicated normal findings; there was no evidence of liver cirrhosis or a tumor. Laboratory tests showed an HCV load of 1.44E + 06 IU/mL and positivity for HCV genotype 1 b. The patient tested negative for human immunodeficiency and hepatitis B viruses. The hemoglobin (Hb) level was 93.0 g/L, the serum aspartate transaminase (AST) was 28 U/L, the alanine transaminase (ALT) concentrations was 15 U/L and the urea clearance index was 1.93/week.

The patient’s past medical history was chronic nephritis, CKD 5 G, anemia, mineral and bone disorder and hyperlipidemia. HD was performed for 17 years, treatment was switched to continuous ambulatory peritoneal dialysis 5 years ago for arteriovenous fistula dysfunction. The patient lost residual renal function during the dialysis. The patient’s medications included erythropoietin, iron, calcium acetate and atorvastatin. The PD prescription was a total volume of 8 L, 6 L of 1.5% PD fluid, and 2 L of 2.5% PD fluid.

HCV treatment was initiated with EBR 50 mg and GZR 100 mg once daily for 12 weeks. Atorvastatin was stopped for drug–drug interactions with EBR/GZR before treatment. Hepatitis C virus RNA, Hb, AST, ALT, albumin (Alb), and total bilirubin (TBIL) were monitored every 4 weeks. A sustained virological response was defined as an HCV RNA level of <15 IU/mL at 12 weeks after the end of treatment.

**Figure 1.**
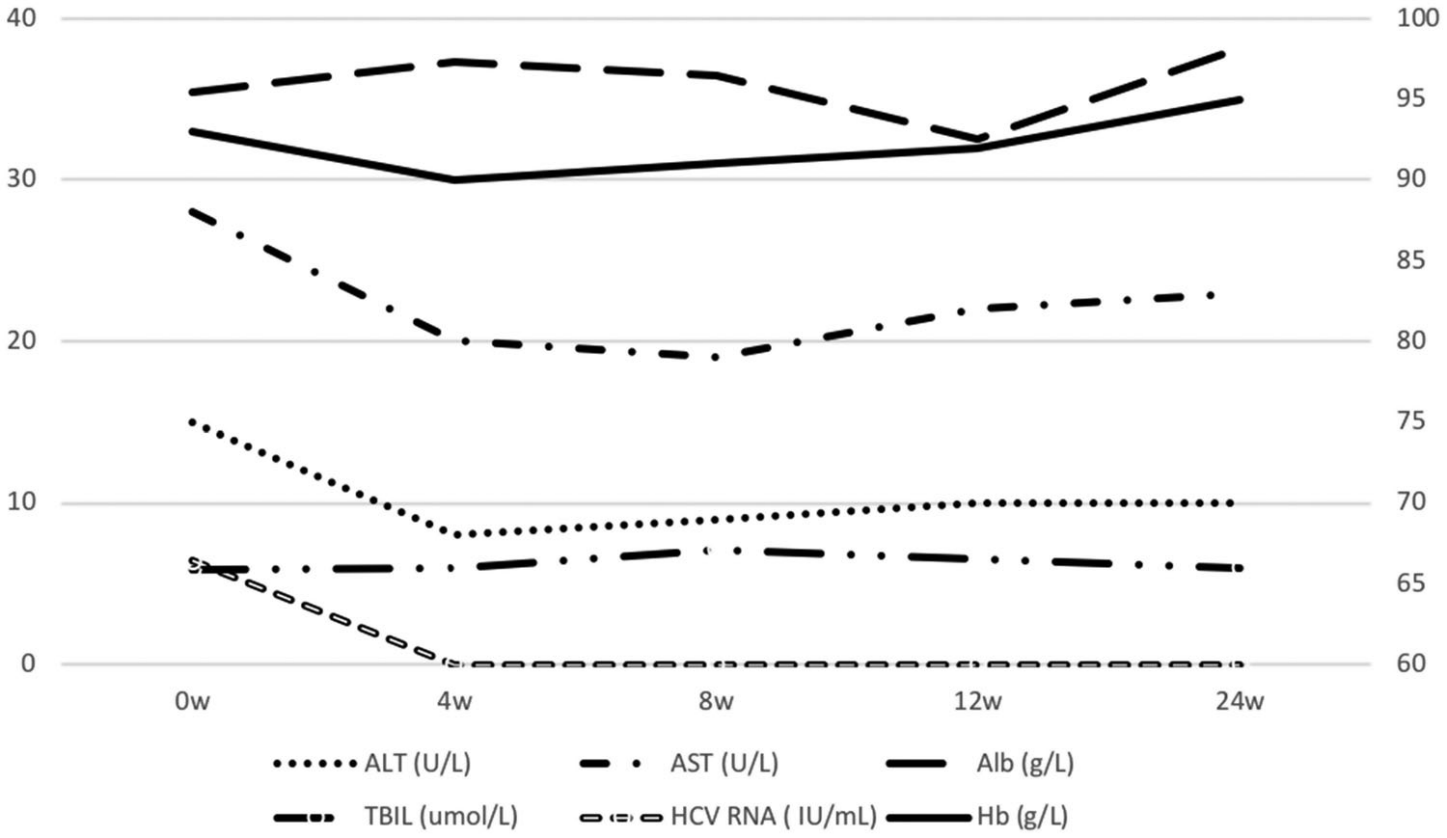
Changes in AST, ALT, Alb, TBIL, and Hb during the treatment.

The treatment was not discontinued due to any adverse event. The patient reported fatigue after starting treatment but it was tolerable. All symptoms resolved after the 12-week treatment completed. The levels of AST, ALT, Alb, TBIL, and Hb were stable (Figure 1). Hepatitis C virus RNA was undetectable in plasma after 4 weeks of treatment, sustained virological response were achieved.

## Discussion

Treatment of HCV-infected dialysis patients can eliminate the infection source, reducing the chances of transmission. There is a significant survival benefit of HCV treatment both in the general population and HD patients [11,12]. The decision to treat HCV in dialysis patients should be based on health benefits, the risks and costs of therapy [13]. Although the possibility of HCV transmission is much lower in PD patients than in HD patients, antiviral therapy should be considered for improving survival and life quality in PD patients. Many of the approved DAAs therapies are not ideal regimens for dialysis patients because they contain drugs that are eliminated by the kidney.

There are very few reports on DAA regimens in PD patients. Aggarwal *et al.* treated HCV-infected dialysis patients with 15 sofosbuvir/velpatasvir courses, one PD patient enrolled. Treatment was generally safe and well tolerated. Patient achieved SVR12 after the therapy [14]. Borgia SM *et al.* used same regimen for dialysis patients, five PD patients included, minor adverse effects were observed in their patients, resulting in a cure rate of 95% [15]. The safety and efficacy of 12-week ombitasvir/paritaprevir/ritonavir treatment in PD patients were evaluated by Shuster *et al.* and Stark *et al.* Intensive pharmacokinetic data showed the individual concentration–time profiles for the drugs overlapped among patients, with considerable inter-patient variability. PD had minimal effects on drugs exposure (area under the curve, AUCs). All of their three PD patients completed treatment and achieved SVR12 [16,17]. Londoño *et al.* described Ombitasvir/paritaprevir/ritonavir and dasabuvir or Ombitasvir/paritaprevir/ritonavir with or without ribavirin (RBV) regimens in CKD patients, included twelve PD patients, no statistically significant differences were found across renal stages. Although anemia, asthenia and pruritus were the most common adverse events during treatment, regimens were also well tolerated in these patients. High SVR12 rate obtained (92.6%) is particularly noteworthy [18]. The above-mentioned reports indicated high cure rates and favorable safety profiles for the drugs in PD patients.

EBR, a nonstructural protein 5 A inhibitor, and GZR, an HCV nonstructural 3/4 A protease inhibitor, can effectively inhibit replication of HCV genotype 1 and 4. The apparent terminal elimination t1⁄2 of both drugs were similar between health group and severe renal impairment group. Both drugs mostly eliminated through feces, with <1% being excreted in urine. Dialysis did not remove any EBR (0%) and removed < 0.5% of GZR from plasma because of high protein binging [19]. Their removal by PD is also unlikely since they are highly bound to plasma protein, as reported in the prescribing information (Merck & Co., Inc.). Although pharmacokinetic analyses show that AUCs are higher in individuals with HD (up to 46% higher compared with individuals with normal kidney function), these changes in exposure to the drugs are not considered clinically relevant [20]. No dosage adjustment of EBR/GZR is required in people with any degree of renal impairment, including those receiving dialysis [19]. The most common adverse events (≥10% frequency) were fatigue, headache, nausea, insomnia, decreased hemoglobin. Others included loss of appetite, asthenia, abdominal pain and dyspepsia [7,21].

Toxicity and intolerable adverse effects were primary concerns of DAAs in CKD patients, especially whom were undergoing dialysis. Since there is no experience about EBR/GZR in PD patients, we followed up the patient closely in order to ensure the safety of treatment. EBR/GZR are substrates of CYP3A, Cytochrome P450 family members. Co-administration with strong CYP3A inducers (such as nifedipine, atorvastatin, simvastatin, amiodarone, commonly used in dialysis patients), it may decreased plasma concentrations and potentially reduced antiviral activity of both agents. We checked combined medication carefully to eliminate the risks of drug–drug interactions during the therapy. The patient tolerated EBR/GZR and only reported treatment-related fatigue (minimal adverse effect). Her Hb level did not decrease, while it was a very common side effect in the C-SURFER study [7]. Her other laboratory parameters including ALT, AST, and TBIL were also stable.

There are limitations of our findings. We did not assess individual drug concentration–time profiles or the EBR/GZR concentrations in the exchanged PD fluid. Moreover, further research on EBR/GZR pharmacokinetics in PD patients is required. In conclusion, EBR/GZR treatment was successful in our PD patient with HCV genotype 1 b infection. This is the first report on the safety and efficacy of EBR/GZR therapy in a PD patient.
